# Case Report: Active tuberculosis infection in CAR T-cell recipients post CAR T-cell therapy: a retrospective case series

**DOI:** 10.3389/fcimb.2023.1147454

**Published:** 2023-05-11

**Authors:** Peiling Zhang, Liang Huang, Miao Zheng, Chao Zhang, Dongyi Wan, Jia Wei, Yang Cao

**Affiliations:** ^1^ Department of Hematology, Tongji Hospital, Tongji Medical College, Huazhong University of Science and Technology, Wuhan, Hubei, China; ^2^ Immunotherapy Research Center for Hematologic Diseases of Hubei Province, Wuhan, Hubei, China; ^3^ Department of Pathology, Tongji Hospital, Tongji Medical College, Huazhong University of Science and Technology, Wuhan, Hubei, China; ^4^ Department of Nuclear Medicine, Tongji Hospital, Tongji Medical College, Huazhong University of Science and Technology, Wuhan, Hubei, China

**Keywords:** immunocompromised, host immunity, tuberculosis, extrapulmonary tuberculosis, chimeric antigen receptor T-cell

## Abstract

High response rates in B-cell malignancies have been achieved with chimeric antigen receptor (CAR) T-cell therapy. Emerging reports indicate a risk of active tuberculosis (TB) with novel immunotherapy for tumors. However, studies of TB in patients post CAR T-cell therapy are limited. In this case series study, we describe five patients with active TB post CD19/CD22 target CAR T-cell therapy alone or following autologous stem cell transplantation (ASCT). One of the patients developed active TB within the first 30 days post CAR T-cell therapy, and fever was the dominant presenting symptom; extrapulmonary manifestations of active TB were common in the other four patients and manifested after the first 30 days of CAR T-cell therapy. Four of the five patients improved with anti-TB treatment, but one patient with isoniazid resistance died of central nervous system TB infection. Our study provides the first series report of active TB following CD19/CD22 target CAR T-cell therapy.

## 1 Introduction

Chimeric antigen receptor (CAR) T-cell therapy has revolutionized the management of relapsed/refractory (r/r) B-cell malignancy. Despite the efficacy of CD19-targeted CAR (CAR19) T-cell therapy, growing numbers of infectious complications have recently been reported in patients receiving this treatment (Hill et al., 2018; Park et al., 2018; Wudhikarn et al., 2020; Logue et al., 2021). The incidence of overall infection in institutional cohorts of patients treated with CAR19 T cells within the first 90 days post CAR T-cell infusion ranges from 23% to 45% (Hill et al., 2018; Wudhikarn et al., 2020; Logue et al., 2021). CAR T-cell recipients are considered as being at increased risk for infection due to underlying malignancy, extensive prior antitumor therapies, and pre-CAR T-cell infusion lymphodepletion chemotherapy (Hill et al., 2018). Furthermore, cytokine release syndrome (CRS), the most common CAR T-cell-related toxicity requiring treatment with corticosteroids and/or tocilizumab, may increase risk of infection (Park et al., 2018). Moreover, CAR19 T cells deplete normal CD19+ B cells in most patients, which contributes to hypogammaglobulinemia.

Our previous work together with other trials suggest that CAR T-cell therapy is safe and effective for r/r B-cell malignancy (Wang et al., 2020; Yan et al., 2022). However, the infection etiologic spectrum of CAR T-cell recipients post CAR T-cell therapy has not been fully clarified. The majority of infection events are bacterial in the first 28 days after CAR T-cell therapy, with respiratory viral infections predominating at later time points (Hill et al., 2018). Severe infection complications, including opportunistic infections involving, e.g., *Pneumocystis jirovecii*, *Varicella Zoster virus*, *Aspergillus, Fusarium*, and *Mucorales*, have been reported (Haidar et al., 2020; Logue et al., 2021). Furthermore, there are emerging reports that risk of active tuberculosis (TB) is increased by PD-1 blockade (Morelli et al., 2022), a novel immunotherapy revolutionizing cancer treatment. Nevertheless, there are scarce data on active TB post CAR T-cell therapy.

In this study, we present five cases of active TB post CD19/CD22 target CAR (CAR19/22) T-cell therapy alone or following autologous stem cell transplantation (ASCT). In view of these cases, we emphasize the need for early screening and diagnosis, comprehensive supportive care, including long-term monitoring and prophylaxis, and treatment management for TB in the era of CAR T-cell therapy.

## 2 Methods

We performed a retrospective electronic medical records review of 427 patients who received CAR T-cell therapy in two trials at Tongji Hospital from January 2017 to December 2021. We included patients diagnosed with active TB after CAR T-cell therapy. Active TB was defined as microbiological tests demonstrating TB in any clinical specimen based on acid-fast bacillus (AFB) staining, mycobacterial culture, Xpert MTB/Rif, or polymerase chain reaction (PCR) for TB, accompanied by clinical and/or radiographic evidence of current disease (Zumla et al., 2013). All five cases of active TB were confirmed. Of the five TB cases, two occurred after CAR19/22 T-cell cocktail therapy (ChiCTR-OPN-16008526) and three after CAR19/22 T-cell cocktail therapy in tandem with ASCT (ChiCTR-OPN-16009847) (shown in **Supplemental Figure 1**).

Clinical, laboratory, imaging, and treatment data for the confirmed cases were collected from the medical record system, including a thorough clinical history of malignancy, history of prior therapy, dose of CAR T-cell infusion, adverse events, and responses after infusion of CAR T-cells. Additionally, TB-related clinical manifestations (e.g., cough, fever, night sweats, weight loss), microbiology studies of TB (e.g., AFB staining, mycobacterial cultures, Xpert MTB/Rif, PCR, T-SPOT.TB test), other relevant laboratory results correlating with immune status and concomitant presence of other infection (e.g., white blood cell (WBC) count, levels of immunoglobulin, inflammatory markers including interleukin-6 (IL-6), high-sensitivity C-reactive protein (hsCRP), and ferritin, lymphocyte subsets), radiographs (e.g., chest X-rays, chest computerized tomography (CT), cranial magnetic resonance imaging (MRI), positron emission tomography/computed tomography (PET/CT)), the process of treatment, and outcome of the patients were recorded for further analysis.

The study was carried out in accordance with the Declaration of Helsinki and approved by the ethics committee of Tongji Medical College, Huazhong University of Science and Technology.

## 3 Results

### 3.1 Baseline characteristics

The baseline characteristics of the patients are shown in **Table 1**. Four patients received CAR T-cell therapy for relapsed or refractory lymphoma and one patient for acute lymphoblastic leukemia. All patients received at least 3 lines of antitumor treatment regimens before CAR T-cell therapy. CRS of grade 1-2 developed and was characterized by fever with or without hypotension and resolved with short-term glucocorticoids or supportive therapy. None of the patients received tocilizumab. No immune effector cell-associated neurotoxicity syndrome (ICANS) was observed. Although neutropenia resolved within three weeks, B-cell aplasia and hypoglobulinemia continued during active TB (**Supplemental Table 1**), and intravenous immunoglobulin was given. CD4+ T cells decreased from normal in the five patients and four patients had a CD4+ T cell count of ≤100 cells/μL during active TB (**Supplemental Table 1**). All patients denied a history of TB or previous known exposure. Because testing for latent TB is not routinely performed among CAR T-cell therapy recipients, the T-SPOT.TB test at baseline was only performed for patient 5, with a positive result. None of the patients had received isoniazid prophylaxis. No coinfection with cytomegalovirus, Epstein-Barr virus, or hepatitis B virus was detected prior to or concomitant with TB presentation in any of the patients based on manifestations and routine specific viral nucleic acid testing.

**Table 1:** Baseline characteristics of patients with tuberculosis .

Patient	Age, Years	Sex	Malignancy	Prior lines of treatment	Conditioning regimen	CAR T-cell dose	In tandem with ASCT	CRS grade	CRS treatment	Time-to neutrophil recovery^†^	Time-to endogenous B-cell recovery^†^
1	45	Male	MCL	7	BEAM	CAR19 T cells 3×10^6^/kg; CAR22 T cells 3×10^6^/kg	Yes	2	Dexamethasone 10 mg per day for 3 days	14 days	3 months
2	29	Female	ALL	3	FC	CAR19 T cells 4×10^6^/kg; CAR22 T cells 4×10^6^/kg	No	1	Supportive	7 days	3 months
3	60	Male	DLBCL	4	BEAM	CAR19 T cells 4.5×10^6^/kg; CAR22 T cells 5.6×10^6^/kg	Yes	1	Supportive	20 days	10 months
4	23	Male	DLBCL	3	BEAM	CAR19 T cells 4.6×10^6^/kg; CAR22 T cells 4.1×10^6^/kg	Yes	1	Supportive	9 days	13 months
5	63	Female	FL	3	FC	CAR19 T cells 2×10^6^/kg; CAR22 T cells 2.7×10^6^/kg	No	1	Supportive	10 days	N/A^*^

^†^Time calculated since CAR T-cell infusion. ^*^ B cells remained significantly depleted at the last follow-up at 12 months post CAR T-cell therapy.

MCL: mantle cell lymphoma; ALL: acute lymphocytic leukemia; DLBCL: diffuse large B-cell lymphoma; FL: follicular lymphoma; BEAM: bis-carmusitine, etoposide, cytarabine and melphalan; FC: fludarabine and cyclophosphamide; CAR: chimeric antigen receptor; CAR19: CD19-targeted chimeric antigen receptor; CAR22: CD22-targeted chimeric antigen receptor; ASCT: autologous stem cell transplantation; CRS: cytokine release syndrome; N/A: not applicable

### 3.2 Clinical presentations and treatment

The clinical presentations of the patients are described in **Table 2**. Patient 1 manifested active TB within the first 30 days after CAR T-cell therapy. He developed a fever up to 39°C on day 4 and had persistent fever along with new hypotension in the ensuing 72 hours; IL-6 peaked at 1044 pg/mL on day 7. Due to grade 2 CRS, he received dexamethasone 10 mg per day for three days and was transitioned from levofloxacin to meropenem and demethovancomycin intravenously. His symptoms improved on day 10, and IL-6 gradually returned to baseline levels on day 12. His neutropenia resolved before day 14. However, low-grade afternoon fever lasted, and the patient developed cough with bloody sputum on day 23. CT scanning demonstrated patchy infiltrates and irregular flaky high-density shadows on his left lung. Xpert MTB/Rif of sputum was positive for rifampin-sensitive *Mycobacterium tuberculosis* (MTB). He had a good response to standard anti-TB therapy, with a normal body temperature.

**Table 2:** Findings and outcomes for patients with tuberculosis.

Patient	Time after CAR T-cell therapy to TB presentation	Initial symptoms	Imaging findings	TB location	Diagnosis	TB Treatment (n, months)	Outcome (Cause)
1	23 days	fever and cough	CT Chest - patchy infiltrates and irregular flaky high-density shadow on left lung.	lungs	Xpert MTB/Rif (sputum)	2HRZE/4HR	Alive
2	66 days	headache, fever, vomiting and cough	CT Chest - diffuse miliary nodules throughout both lungs; Cranial MRI - focal FLAIR-weighted signal hyperintensity in left posterior limbs of the internal capsules, left corona radiata and right parietal lobe.	lungs and CNS	Xpert MTB/Rif (CSF)	2HRZE	Relapse of ALL after 4 months of CAR T-cell therapy and lost to follow-up
3	8 months	difficulty breathing, cough, night sweats and weight loss	CT Chest - massive pleural effusion and multiple small lung nodules.	lungs and CNS	Xpert MTB/Rif (PE); TB PCR (CSF)	2HRZE^†^	Death (coma and respiratory circulatory failure due to CNS TB)
4	10 months	cough	PET/CT - nodules and patches at the right lower lobe with a SUVmax of 10.6 and enlargement of multiple mediastinal and hilar lymph nodes with a SUVmax of 17.5.	Lungs and mediastinal lymph nodes	TB PCR (mediastinal lymph node biopsy)	3HRZE/9HER	Alive
5	11 months	painless subcutaneous malar nodules	PET/CT - a hypermetabolic subcutaneous malar foci with a SUVmax of 17.9 and multiple intense FDG uptake of skull and T3 vertebra with SUVmax ranging from 12.6 to 15.9.	subcutaneous tissue and bone	TB PCR (subcutaneous nodules biopsy)	3HRZE/9HER	Alive

^†^Patient 3 discontinued anti-TB treatment after 2 months of his own accord. After 5 months of withdrawal, he was diagnosed with central nervous system tuberculosis with isoniazid resistance and received RZ, levofloxacin, and linezolid as anti-TB treatment.

CAR: chimeric antigen receptor; TB: tuberculosis; CT: computerized tomography; MRI: magnetic resonance imaging; PET: positron emission tomography; fluorodeoxyglucose; SUV: standardized uptake value; FDG: fluorodeoxyglucose; CNS: central nervous system; CSF: cerebrospinal fluid; PE: pleural effusion; H: isoniazid; R: rifampin; Z: pyrazinamide; E: ethambutol

**Figure 1:**
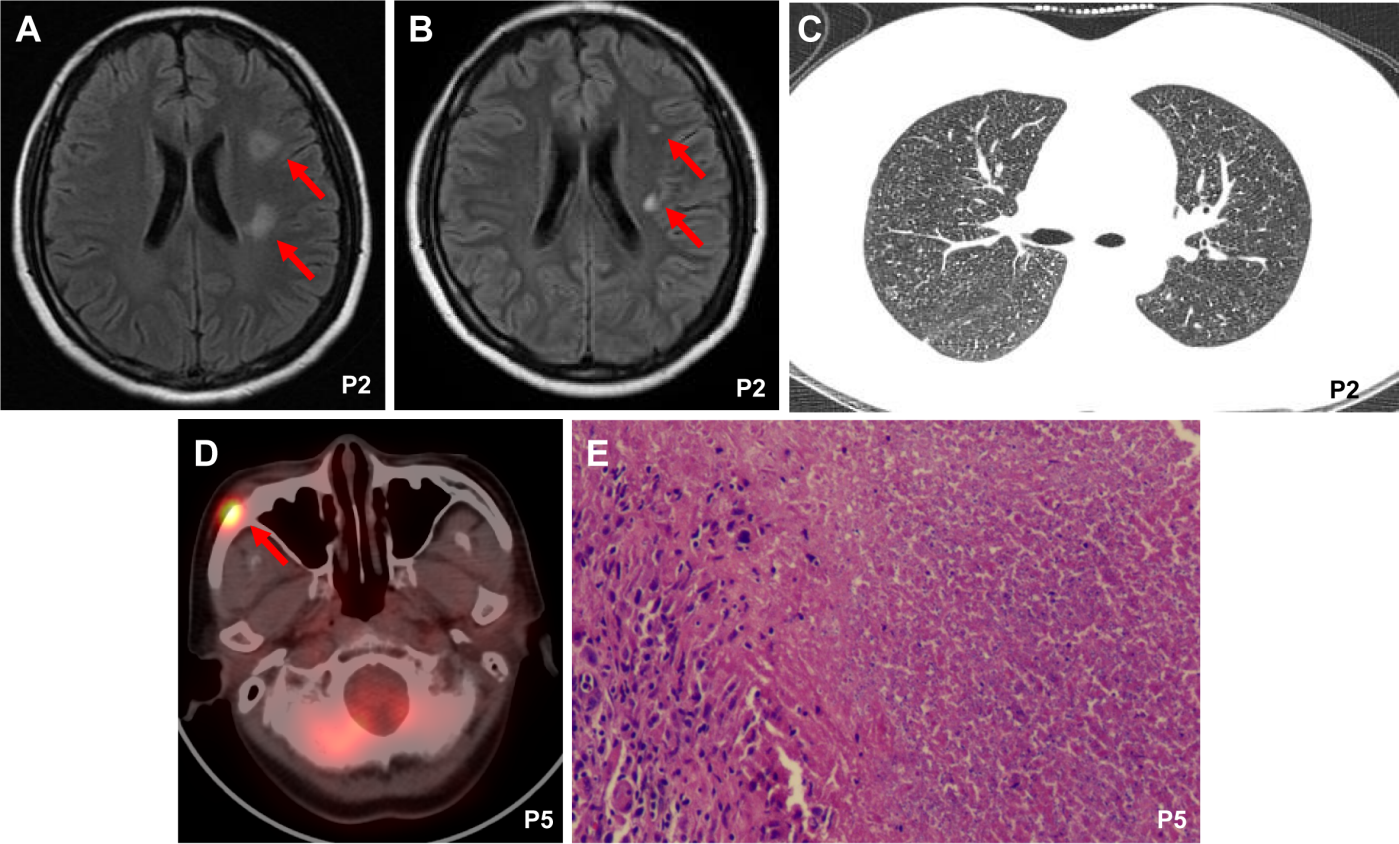
Imaging findings and tissue biopsy. **(A)** Cranial magnetic resonance imaging (MRI) showed focal FLAIR-weighted signal hyperintensity in patient 2 after 2 months of CAR19/22 T-cell cocktail therapy**. (B)** The MRI image of patient 2 after anti-TB therapy for one month. **(C)** Chest computerized tomography (CT) images showed disseminated miliary nodules in bilateral lung fields in patient 2 after 2 months of CAR19/22 T-cell cocktail therapy. **(D)** Positron emission tomography/computed tomography (PET/CT) scanning of patient 5 after 11 months of CAR19/22 T-cell cocktail therapy. **(E)** Pathology of the subcutaneous nodules on the right malar region in patient 5 revealed necrotizing granuloma. P, Patient.

In our case series, extrapulmonary manifestations of TB occurred after the first 30 days of CAR T-cell therapy. Patient 2 presented with symptoms of headache, fever, vomiting, and cough from day 66 after CAR T-cell therapy, and a cerebrospinal fluid (CSF) sample was positive for Xpert MTB/Rif at this time. Cranial magnetic resonance imaging (MRI) showed focal FLAIR-weighted signal hyperintensity in the left posterior limbs of the internal capsules, left corona radiata, and right parietal lobe (**Figure 1A**). CT revealed disseminated miliary nodules in bilateral lung fields (**Figure 1C**). Patient 5 presented with painless subcutaneous nodules on the right malar region after 11 months of CAR T-cell therapy, which displayed intense fluorodeoxyglucose (FDG) uptake on [(18)F]-FDG positron emission tomography/computed tomography (PET/CT) scanning (**Figure 1D**). Pathology of the nodule indicated necrotizing granuloma (**Figure 1E**), and MTB DNA was detectable by PCR. Although patient 3 was diagnosed with TB based on Xpert MTB/Rif of pleural effusion, he discontinued anti-TB treatment after 2 months of his own accord. At 5 months after withdrawal, he was admitted to the ICU of a local hospital with a 1-day history of coma after headache and fever for 20 days. Analysis of CSF samples was positive for MTB DNA by PCR and revealed a -15C>T mutation in the inhA promoter (Vilchèze and Jacobs, 2014), which has a strong association with isoniazid resistance. His CSF culture was negative.

Patient 4 developed mild cough at 10 months after CAR T-cell therapy. PET/CT scanning showed newly emerged nodules and patches at the right lower lobe and enlargement of multiple mediastinal and hilar lymph nodes with intense FDG uptake at routine follow-up examinations of CAR T-cell therapy. Metastatic lymph node samples from patient 4 obtained *via* endobronchial ultrasound-guided transbronchial needle aspiration (EBUS-TBNA) were pathologically diagnosed as chronic granulomatous inflammation. The MTB PCR results were positive.

All patients received first-line quadruple drug intensive therapy with isoniazid (H), rifampin (R), pyrazinamide (Z), and ethambutol (E) for two months and continuation therapy for at least 4 months, without TB recurrence, except for patient 2 and patient 3. Patient 2 showed improvement of both symptoms and imaging after treatment for one month (**Figure 1B**). However, acute lymphoblastic leukemia recurred after 4 months of CAR T-cell therapy and 2 months of anti-TB treatment, and the patient was lost to follow-up. Patient 3 received RZ, levofloxacin, and linezolid as anti-TB treatment and glycerin to control increased intracranial pressure after drug resistance was detected. Unfortunately, the patient experienced respiratory circulatory failure and eventually died at 12 days after admission.

## 4 Discussion

While wide-scale application of CAR T-cell therapy for B-cell malignancies has shown unprecedented success, adverse effects of CAR T-cell therapy are now appearing, including infectious complications. Indeed, CAR T-cell recipients are at increased risk of infection due to immune deficiencies caused by previous immunosuppression, lymphodepleting chemotherapy, treatment with tocilizumab and/or steroids, on-target effects of hypogammaglobulinemia, and protracted cytopenia (Bupha-Intr et al., 2021). The most common infections are bacterial, viral, and fungal infection (Hill et al., 2018; Park et al., 2018; Vora et al., 2020). Although many recommendations are based on other hematological treatment modalities, screening, monitoring, and prophylaxis might not be sufficient for CAR T-cell recipients, and there is a lack of clinical trials to define how to screen, monitor, and prevent infection effectively.

TB accounts for millions of deaths worldwide and is very harmful, particularly in patients with immune deficiencies (Pai et al., 2016). Immune deficiencies increase risk of active TB and mortality due to high incidences of extrapulmonary and disseminated TB (Meintjes et al., 2019; Bergeron et al., 2022). In addition, diagnosing TB is difficult owing to the high incidences of extrapulmonary TB and the low sensitivity of the tuberculin skin test (TST) and interferon gamma release assays (IGRA) for immunocompromised patients (Meintjes et al., 2019; Bergeron et al., 2022). Overall, risk of active TB increases soon after infection with human immunodeficiency virus (HIV), and one in five HIV-related deaths are due to TB (Zumla et al., 2013; Pai et al., 2016). In this study, we describe five cases of active TB in CAR T-cell recipients with B-cell malignancies. To our knowledge, this is the first report of a case series in adults, though one TB case in a child with acute lymphoblastic leukemia following CAR T-cell therapy has recently been reported (Grasa et al., 2022). Symptoms and chest imaging indicative of active TB were screened before CAR T-cell therapy in our center. This study indicates that more proactive screening and prophylaxis for TB may be necessary prior to CAR T-cell therapy. Currently, there is a lack of consensus on the best strategy for TB screening and prophylaxis in CAR T-cell recipients. Nonetheless, there are some recommendations based on cancer patients, including that screening for TB, including TST and IGRA, should be performed prior to CAR T-cell therapy in patients with risk factors for exposure, particularly in TB-endemic regions (Hill and Seo, 2020). Prophylaxis for active TB is controversial, and there are few data regarding patients with hematological malignancies (Bergeron et al., 2022). The optimal duration and time to start prophylaxis should be determined based on large-sample, prospective clinical trials.

The link between CAR-T cells and infection and its mechanism are unclear. We collected lymphocyte subsets and levels of immunoglobulins during active TB. Notably, CD4+ T-cell lymphopenia was observed in most CAR T-cell recipients in our case series, accompanied by B-cell aplasia and hypoglobulinemia. Some studies have found profound and prolonged CD4 T-cell immunosuppression for more than one year in CAR T-cell recipients, with a long-term risk of infectious complications (Logue et al., 2021; Strati et al., 2021). Protective immune responses against *Mycobacterium tuberculosis* are largely mediated by CD4+ Th1 cells, which secrete IFN-γ (O’Garra et al., 2013). CD4+ T-cell lymphopenia is a risk factor for progression from latent TB to active TB (Pai et al., 2016), which may be associated with an increased risk of active TB in CAR T-cell recipients. Furthermore, CD4 counts of less than 200 per cubic millimeter may increase the presentation of extrapulmonary TB (Zumla et al., 2013), which was observed in four of the five patients in this study. A reduction in CD4 counts may suggest the timing of TB prophylactic treatment.

There are limitations to our study. We retrospectively collected CAR T-cell recipients with positive TB microbiological tests at a single center, with only a small sample size of patients with active TB. This brings difficulties with regard to calculating the exact risk of TB in CAR T-cell recipients. As commercial use of CAR T-cell products and an increasing number of CAR T-cell clinical trials are ongoing, more data should be collected to assess the risk of TB in CAR T-cell recipients. The timing and role of TB prophylactic treatment or preemptive treatment after CAR T-cell therapy should also be defined in detail.

## Data availability statement

The raw data supporting the conclusions of this article will be made available by the authors, without undue reservation.

## Ethics statement

The studies involving human participants were reviewed and approved by Ethics committee of Tongji Medical College, Huazhong University of Science and Technology. The patients/participants provided their written informed consent to participate in this study.

## Author contributions

PZ, JW, and YC analyzed the data and wrote the manuscript. LH and MZ managed the patients. PZ, CZ, and DW collected the data. JW and YC revised the manuscript and were in charge of the final approval of the manuscript. All authors read and approved the final manuscript.
