# Supplementary material for: Case Report: Active tuberculosis infection in CAR T-cell recipients post CAR T-cell therapy: a retrospective case series
Source: Front Cell Infect Microbiol. 2023 May 11;13:1147454. doi: 10.3389/fcimb.2023.1147454 (PMC10210154; doi:10.3389/fcimb.2023.1147454)
Supplement: Supplementary file 1 [file DataSheet_1.docx]

Supplementary Material

Active Tuberculosis Infection In CAR T-Cell Recipients Post CAR T-Cell Therapy：A Retrospective Case Series

Peiling Zhang*， Liang Huang, Miao Zheng, Chao Zhang, Dongyi Wan, Jia Wei, Yang Cao

*** Correspondence:** Yang Cao: caoyangemma@163.com, Jia Wei: [jiawei@tjh.tjmu.edu.cn](mailto:jiawei@tjh.tjmu.edu.cn)

# Supplementary Figures and Tables

## Supplementary Figures

**
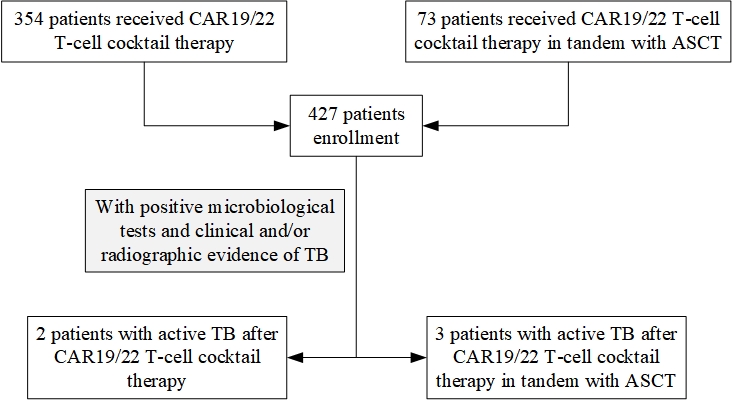
**

**Supplementary Figure 1. Annotated flow diagram of patients included in the study.**

## Supplementary Table s

**Supplementary Table 1. Immune status of CAR T-Cell Recipients during active TB.**

| **Patient** | **Lymphocyte count (cells/μL)** | | | |  | **Immunoglobulin (g/L)** | | |
| --- | --- | --- | --- | --- | --- | --- | --- | --- |
|  | **CD3+CD4+**  **(Range: 550–1440)** | **CD3+CD8+**  **(Range: 320–1250)** | **CD3-CD19+ (Range: 90–560)** | **CD3-CD16+CD56+**  **(Range: 150–1100)** |  | **IgG**  **(Range: 7.51–15.6)** | **IgM**  **(Range: 0.46–3.04)** | **IgA**  **(Range: 0.82–4.53)** |
| 1 | 23↓ | 231↓ | 0↓ | 4↓ |  | 3↓ | 0.1↓ | <0.07↓ |
| 2 | 92↓ | 148↓ | 0↓ | 60↓ |  | 3.6↓ | 0.14↓ | 0.13↓ |
| 3 | 74↓ | 428 | 0↓ | 15↓ |  | 3.7↓ | <0.04↓ | <0.07↓ |
| 4 | 96↓ | 201↓ | 12↓ | 83↓ |  | 4.5↓ | 0.3↓ | 0.88↓ |
| 5 | 505↓ | 771 | 0↓ | 56↓ |  | 3↓ | 0.06↓ | <0.07↓ |
